# Optimizing Encapsulation of Active Compounds of Carrot By-Product in TPP-Chitosomes

**DOI:** 10.3390/foods13162604

**Published:** 2024-08-20

**Authors:** Elisa Malagutti, Sabrina Guarda Botelho Pinho, Marcelo Thomazini, Delia Rita Tapia-Blácido, Milena Martelli Tosi

**Affiliations:** 1 Department of Chemistry, Faculty of Philosophy, Sciences and Letters of Ribeirão Preto, University of São Paulo, Av. Bandeirantes, 3900, Ribeirão Preto CEP 14040-901, São Paulo, Brazil; malaguttielisa@usp.br (E.M.);; 2 Department of Food Engineering, Faculty of Animal Science and Food Engineering, University of São Paulo, Rua Duque de Caxias Norte 225, Pirassununga CEP 13635-900, São Paulo, Brazil; sabrinaguarda@usp.br (S.G.B.P.); mthomazini@usp.br (M.T.)

**Keywords:** encapsulation, by-product, chitosan, optimization, stability, *Daucus carota*

## Abstract

Liposomes coated with chitosan by ionic gelation with tripolyphosphate (TPP-chitosomes) are interesting particles for stabilizing active compounds. However, the encapsulation condition must be optimized. The aim of this study was to optimize the encapsulation of phenolics and carotenoids of carrot pomace in TPP-chitosomes by using a Central Composite Design 2^3^ and response surface methodology. The independent variables were the phospholipid (0.8–4.2 mg/mL), chitosan (2.6–9.4 mg/mL), and carrot pomace (4–14 g/100 mL of ethanol) concentrations; the responses were the encapsulation efficiency in TPP-chitosomes (EE) of phenolics, a-carotene, and b-carotene and the particle size and zeta potential of the particles. The zeta potential ranged from +17 to +37 mV, indicating that the liposomes were coated with chitosan and that the particle sizes were in the nanometric to submicrometric scale. The optimized condition for encapsulating carotenoids was 2.5 mg/mL phospholipids, 6.0 mg/mL chitosan, and 12 g of carrot pomace/100 mL of ethanol. In this condition, the EE of phenolics and α- and β-carotene was 95%, 98%, and 99%, respectively. Therefore, TPP-chitosomes containing encapsulated phenolics and carotenoids, which can be obtained from agro-industrial by-products, have potential application as natural pigments in food or cosmetics. TPP-chitosomes can also be used to encapsulate other types of natural pigments.

## 1. Introduction

Fruits and vegetables deteriorate fast. Processing food by drying, cooking, or refrigeration is a valuable tool to increase the shelf life of these perishable products. Despite advances in food processing techniques, high volumes of waste are generated worldwide. According to a report published by the United Nations Environment Program, around 931 million tons of food were wasted in 2019, which corresponds to approximately 17% of the total food produced in the world [1]. Approximately one-third of the industrial food intended for human consumption is wasted annually, with 38% of this wastage occurring during the food processing stage [2]. Alternatives are being sought to minimize this waste, especially when it comes to industrially generated by-products, which exhibit important nutraceutical properties and contain bioactive molecules [3]. Many studies have been devoted to recovering such molecules and incorporating them into new foods, to provide healthier and more functional foods [4,5].

Carrots (*Daucus carota*) belong to the group of tuberous roots, are the main root vegetable in terms of economic value, and are among the ten most cultivated vegetable species in Brazil [6]. The global production of carrots has seen a steady increase, driven by rising demand for healthful and nutritious foods [7]. In 2021, global carrot and turnip production reached around 42 million tons [8], reflecting its popularity and importance in diets across various cultures. In addition to having a highly appreciated flavor, carrots are a source of fiber, vitamins, phenolics, carotenoids, and other bioactive compounds, which benefit consumers’ health [9]. Lutein and α- and β-carotene occur in orange carrots, whose color is due to α- and β-carotenoids [10]. Although carrots are widely consumed, a large volume are discarded mainly because they do not meet market standards. Large-scale commercial carrot processors are estimated to waste about 175,000 tons of this vegetable per year [11,12]. To minimize waste and financial loss, some agricultural sectors have used carrot waste to feed animals or to produce juice and have employed minimal processing to obtain baby carrots, matchsticks, and shredded carrots for human consumption. However, these processes generate by-products in the form of mash or pomace, which are edible and nutrient-dense [11]. For example, these by-products contain phenolics and carotenoids, mainly α- and β-carotene [13], which play a key role in vitamin A production. They are also frequently used as a food additive to color foods, a practice that has been employed for centuries [14] and remains common today [15]. Unfortunately, these bioactive compounds are extremely sensitive and unstable in industrial conditions of temperature and pH and in the presence of light or oxygen [4], which limits their application in the food industry. Encapsulation of plant pigments emerges as an interesting solution to address these issues [16].

Phenolic compounds generally play an important role in the flavor, color, and physical–chemical properties of foods, affecting their quality and stability. Strong interest has been observed in the use of polyphenols to modify the properties of certain ingredients, such as proteins and lipids, to improve the quality and nutrition of foods [17,18]. Carotenoids, in particular, β-carotene, are an important biological component, being a precursor to vitamin A, also known as retinol. As humans are not capable of synthesizing carotenoids, their consumption through a balanced diet becomes essential. Approximately 10 to 15% of the total β-carotene consumed in food is absorbed in the intestinal mucosa, being partially converted into vitamin A through enzymatic reactions [19,20].

Although studies indicate that a wide range of phytochemicals have anticarcinogenic and antioxidant activities, through mechanisms that involve the direct elimination of free radicals, metal chelation, and inhibition of proteases [21,22,23], these compounds are very sensitive and chemically unstable [24]. Studies indicate that many phytochemical compounds are sensitive to heat and can degrade or lose part of their biological activity at high temperatures [25] and can also degrade over time [26].

Considering these challenges, techniques have been developed to minimize the effects of external parameters such as temperature, light, enzymatic action, and oxygen on the degradation of these compounds [27]. Nanoencapsulation is a promising technique in food science and technology, offering several benefits, including the protection and stabilization of these compounds [28], solubility and bioavailability improvement, and controlled release [29].

Nanoencapsulation involves the encapsulation of compounds in nanometric structures, such as liposomes, micelles, nanoparticles, and nanofibers. Liposomes, small biocompatible vesicles consisting of at least one phospholipid layer, are spontaneously formed in aqueous solutions. In the food area, liposomes have been investigated for encapsulating sensitive active compounds, to protect them against degradation. Given that liposomes are amphiphilic, they can co-encapsulate compounds of different polarities [30]. Nevertheless, liposomes are thermodynamically unstable and release bioactive compounds in a non-controlled way. Studies have shown that coating liposomes with biopolymers (for instance, chitosan) stabilizes them and forms a structure known as chitosomes [31,32,33], which can be easily incorporated into aqueous suspensions, such as biodegradable films [34]. Chitosan is an interesting biopolymer due to its biocompatibility, low toxicity, and the presence of positively charged amino groups in aqueous suspension, thereby increasing particle stability.

At the appropriate concentration and conditions, chitosan chains can be adsorbed on the surface of the vesicles, generating a layer that can vary between 20 and 100 nm [35]. This layer can create physical barriers and prevent liposomes from aggregating, in addition to forming a dense mesh covering that prevents external agents with negative charges, such as oxygen ions, from approaching [36]. Numerous studies have compared liposome particles with chitosomes and concluded that chitosomes are more stable and show better inhibition effects on the degradation of encapsulated bioactive compounds [31,32,33,37,38]

Tripolyphosphate (TPP) chitosan-based nanoparticles offer several advantages in the food industry. They can enhance the antimicrobial properties of food products by inhibiting the growth of a wide range of microorganisms, thereby extending the shelf life of these products [39]. Additionally, they can be utilized in intelligent food packaging systems to improve food quality and safety [40]. These particles can also be incorporated into new foods for encapsulating bioactive compounds, enabling controlled release and the creation of nutritionally enriched foods.

Recent studies have proposed adding a crosslinking agent (e.g., sodium tripolyphosphate) during chitosan complexation with liposomes to stabilize the resulting chitosomes even further [31,33,37,38]. However, few studies have focused on the co-encapsulation of different compounds. Esposto et al. [33] co-encapsulated phenolics and carotenoids of carrot by-product in TPP-chitosomes and obtained an encapsulation efficiency (EE) of 86%, 76%, and 79% for phenolics and α- and β-carotene, respectively. Moreover, these authors did not verify how the concentrations of the particle constituents (phospholipid and chitosan) or by-products affect the resulting particles. In this context, the main objective of this study was to evaluate how the phospholipid, chitosan, and carrot pomace concentrations affect the characteristics of the resulting TPP-chitosomes containing encapsulated bioactive compounds, to optimize the efficiency of co-encapsulating phenolics and carotenoids in these particles.

## 2. Materials and Methods

### 2.1. Materials

Orange carrots (*Daucus carota* L.) were purchased at a local market (Pirassununga, São Paulo, Brazil), without the presence of leaves. The solvents used, methanol and ethanol P.A. 99.5%, were purchased from Êxodo Científica (São Paulo, Brazil). Gallic acid, α-carotene and β-carotene standards, and reagents sodium carbonate and methyl tert-butyl ether were purchased from Sigma-Aldrich (St. Louis, MI, USA). The Folin–Ciocalteu reagent was purchased from the company Dinâmica Química Contemporânea (Indaiatuba, São Paulo, Brazil). The phospholipids (Phospholipon—P90G, containing over 94% phosphatidylcholine, derived from soy) were obtained from the company Lipid Ingredients (Ribeirão Preto, São Paulo, Brazil). Chitosan of medium molecular weight (Product 448877, MW ranging from 190,000 to 310,000 g/mol, containing 75–85% deacetylated units), sodium tripolyphosphate (MW of 367.86 g/mol), and acetic acid PA were purchased from Sigma-Aldrich (St. Louis, MI, USA).

### 2.2. Extraction and Quantification of Phytochemical Compounds

To obtain the by-product, fresh carrots were thoroughly washed and processed using a home multiprocessor (Juicer XL Philips Walita, Barueri, Brazil). The resulting carrot pomace was then weighed into glass beakers in varying amounts of 4, 6, 9, 12, and 14 g. Each sample was mixed with 100 milliliters of 99.5% ethanol and submitted to ultrasound in a probe sonicator (40% amplitude, 550 W for 11 min, Sonifier^®^ SFX550, Branson Ultrasonics, Danbury, CT, USA, USA). An ice bath was used to avoid overheating and possible degradation of phytochemical compounds, so that the temperature did not exceed 40 °C. Samples were produced in triplicate and filtered through filter paper, obtaining clear ethanolic carrot pomace extracts and stored under refrigeration (4–8 °C).

To measure the total content of phenolics in the samples, the Folin–Ciocalteu method was employed following the methodology proposed by Esposto et al. [33]. The samples, obtained after the extraction process, were diluted 10 times and 500 μL was transferred to test tubes to which 2.50 mL of commercial Folin–Ciocalteu reagent diluted 1:10 (*v*/*v* in distilled water) was added. The tubes were shaken and kept in the dark for 5 min. After this time, 2.00 mL of 4% sodium carbonate solution was added, the tubes were shaken again and kept in the dark for 45 min. Absorbance was read at 760 nm (Shimadzu UV-1601PC; Shimadzu, Kyoto, Japan); the blank was used as a reference. TPC was determined from a gallic acid standard curve (0 to 50 mgL^−1^): y = 13,587x + 0.0188 (R^2^ = 0.9991), and results are expressed as mg of Gallic Acid Equivalent (GAE).

For the quantification of carotenoids, all the samples were filtered and analyzed by High-Performance Liquid Chromatography (HPLC Prominence, Shimadzu, Kyoto, Japan) according to the methodology described previously [33]. Chromatograms were acquired at 450 nm, and data were collected with the LC Solution software (Shimadzu Corporation), version 1.21. Calibration curves previously constructed with external commercial standards (α- and β-carotene) were used to identify and quantify the carotenoids.

### 2.3. Optimization of the Encapsulation of Phytochemical Compounds from Carrot Pomace in TPP-Chitosomes

Carotenoid- and phenolic-rich extracts obtained from carrot pomace were encapsulated in liposomes coated with chitosan by ionic gelation in the presence of sodium TPP, to obtain TPP-chitosomes containing encapsulated phenolics and carotenoids [33]. A Central Composite Design (CCD) 2^3^ (8 experiments + 6 axial points + 3 central points) and response surface methodology (RSM) were used to optimize encapsulation. Three variables—phospholipid (X_1_: 0.8, 1.5, 2.5, 3.5, and 4.2 mg/mL), chitosan (X_2_: 2.6, 4, 6, 8, and 9.4 mg/mL), and carrot pomace (X_3_: 4, 6, 9, 12, and 14 g/100 mL of ethanol) concentrations—were selected to establish the optimized condition. 17 runs were performed for the experimental design (−1.68, −1, 0, +1, and +1.68) (Table 1). Minimum and maximum concentrations had been determined in preliminary tests. All the suspensions were produced in duplicate. The evaluated responses were the EE of phenolics (y_1_), EE of α- (y_2_), EE of β-carotene (y_3_), the particle size (y_4_), and zeta potential (y_5_) of the resulting TPP-chitosomes containing encapsulated phenolics and carotenoids.

To obtain TPP-chitosomes containing encapsulated phenolics and carotenoids, P90G phospholipids were added to an ethanolic carrot pomace extract (100 mL), according to the concentration presented in Table 1, and stirred magnetically for 10 min. Ethanol was completely evaporated using a rotary evaporator at 40 °C (Marconi MA 120, Piracicaba, Brazil). Afterward, 50 mL phosphate buffer saline (pH 7.4) was added, and the suspension was subjected to sonication (5 s on and 2 s off for 25 cycles) to produce liposomes. A chitosan solution was first prepared by solubilizing chitosan in acetic acid solution (1%) for 16 h (pH~3.6). An amount of 50 mL of chitosan solution (at concentrations presented in Table 1) was then added to the suspension of liposomes and maintained under magnetic stirring for 10 min. For ionic gelation, 100 mL of TPP solution (chitosan: TPP 3:1, according to chitosan concentration Table 1) was added dropwise with a burette, to form TPP-chitosomes (pH~4.0). The drip rate was approximately 1 drop per second.

### 2.4. Characterization of TPP-Chitosome Nanoparticles Encapsulating Phytochemical Compounds from Carrot Pomace

#### 2.4.1. Particle Morphology, Size, and Zeta Potential

Morphological analyses were performed by Transmission Electron Microscopy (TEM) according to Esposto et al. [33]. Samples were deposited on a carbon-coated grid, stained with 1.5% uranyl acetate (aqueous solution), dried at room temperature for 24 h, and examined with a JEM 100CXII microscope (Tokyo, Japan) at an accelerating voltage of 80 kV.

Hydrodynamic diameters were measured by the dynamic light scattering (DLS) technique on a Zetasizer Nano ZS Instrument (Malvern Panalytical, Malvern, United Kingdom) [32]. Zeta potential was also obtained on a Zetasizer Nano ZS Instrument. All the measurements were made in triplicate.

#### 2.4.2. Encapsulation Efficiency of Phenolics and β- and α-Carotene

EE was determined according to the methodology proposed by Tan, Feng, Zhang, Xia, and Xia [38], with some modifications. Firstly, the total content of phenolics and the contents of α- and β-carotene were determined. To quantify α- and β-carotene, the sample was centrifuged at 14,000 rcf and 4 °C for 5 min. Then, 1 mL of supernatant was removed and poured into a duly identified empty Eppendorf. Next, 0.7 mL of petroleum ether was added to the Eppendorf, which was vortexed and sonicated. The supernatant was collected and poured into vials, and petroleum ether was evaporated for future quantification of α- and β-carotene by HPLC.

To determine the total content of phenolics, the sample was centrifuged at 7195 rcf and 4 °C for 5 min on Amicon Ultra-0.5 (Amicon Ultra-0.5 mL 3 K device, Merck KGaA, Darmstadt, Germany) to separate phenolics. The filtered solution was used to quantify TPC by the Folin–Ciocalteu colorimetric method.

Finally, EE was calculated as the difference between the total content of bioactive compound in the encapsulated extract (*B_T_*) and the content of bioactive compound in the supernatant (*B_s_*), according to Equation (1).

(1) $$EE(\%)=\frac{B_{T}-B_{S}}{B_{T}}\times 100$$

### 2.5. Statistical Analysis to Optimize Encapsulation of Phytochemical Compounds from Carrot Pomace

RSM is a statistical tool that plays an essential role in visualizing how independent (factors) and response variables (response) are related. RSM uses second-order mathematic models that include linear, interaction, and quadratic effects (Equation (2)) [41]:

(2) $$y=\beta_{o}+\sum_{i=1}^{k}\beta_{i}x_{i}+\sum_{i=1}^{k}\beta_{ii}x_{i}^{2}+\sum_{\begin{array}{c} i=1\\ i=1 \end{array}}^{k}\beta_{ij}x_{i}x_{j}+\varepsilon$$

where *y* is the predicted mean response, *x_i_* and *x_j_* represent independent coded variables that affect the response variable, *β_o_* is the constant term of the model, *β_i_* are linear term coefficients, *β_ii_* are quadratic term coefficients, *β_ij_* are interaction term coefficients, and *ε* is the random error component that is determined by fitting the model to the data.

Data were statistically analyzed with the Statistica^®^ v7 software (Statsoft Inc., Tulsa, OK, USA). Effect analysis, analysis of variance (ANOVA), and the regression equation were obtained at a significance level of *p* < 0.01. The mathematic model is considered significant and predictive when the regression is significant (F_Regression_ > F_tab_, *p*-value < 0.01 mathematic model) and does not present a lack of fit (F_lack of fit_ < F_tab_, *p*-value > 0.01, lack of fit is not significant) [42]. Thus, the mathematic models of responses that were significant and predictive were used for optimization by multiple responses; desirability functions with values restricted to the interval between 0 and 1, where 0 is the least acceptable value, and 1 is the most desirable value [43].

## 3. Results and Discussion

### 3.1. Active Contents of Extracts, the Visual Aspect of TPP-Chitosomes Containing Encapsulated Phenolics and Carotenoids

The results obtained for the extraction of TPC, α-carotene, and β-carotene with different concentrations of by-products are presented in Table 2. The highest TPC content was observed when using a concentration of 12 g/100 mL ethanol (21.1 μg GAE/mL or 175 μg GAE/g of by-product), rather than 14 g/100 mL ethanol (20.0 μg GAE/mL or 143 μg GAE/g of by-product). Among the many parameters that can influence the extraction process, the effect of the solid-to-solvent ratio is significant for extraction efficiency [44]. An adequate amount of solvent should be used to prevent the solution from becoming saturated with the added by-products [45]. Less efficient extractions in samples with higher by-product concentrations may result from the saturation of the extracting solvent.

Esposto et al. [33] studied various conditions for the simultaneous extraction of TPC and carotenoids, varying the solvent concentration, ultrasound time, and by-product concentration. The TPC content found using a by-product concentration of 6 g/100 mL ethanol was 81 μg GAE/g by-product, which is lower than the result found in the present study, where the TPC content was approximately 200 μg GAE/g by-product, equivalent to 12.1 μg GAE/mL of extract (Table 2). This difference could be attributed to plant variability.

Regarding the carotenoid content, the lowest by-product concentration (4 g/100 mL ethanol) also revealed the lowest levels of α and β-carotenes. The by-product concentration of 9 g/100 mL ethanol showed higher results compared to the others. As explained for TPC, the highest by-product concentration did not show higher levels of α and β-carotenes, which can also be attributed to the solvent saturation.

**Figure 1:**
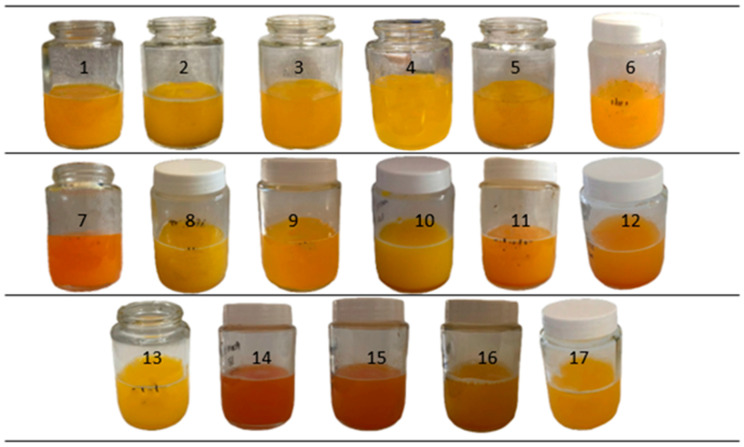
Suspensions of TPP-chitosomes containing encapsulated phenolics and carotenoids prepared according to a CCD 2^3^ experimental design. 1 to 17 refer to samples prepared according to the different concentrations of phospholipids, chitosan and carrot pomace described in Table 1.

Figure 1 shows the suspensions of TPP-chitosomes containing encapsulated phenolics and carotenoids prepared according to a CCD 2^3^ experimental design (Table 1). All the suspensions were homogenous and colorful and had shades of yellow and orange.

TPP-chitosomes containing encapsulated phenolics and carotenoids prepared according to runs 6, 7, 14, 15, and 16 (Table 1) presented stronger orange shades. Phenolics and carotenoids underlie the color of these suspensions, demonstrating that it was possible to extract these bioactive compounds from carrot pomace.

### 3.2. Encapsulation Efficiency of Phenolics

**Figure 2:**
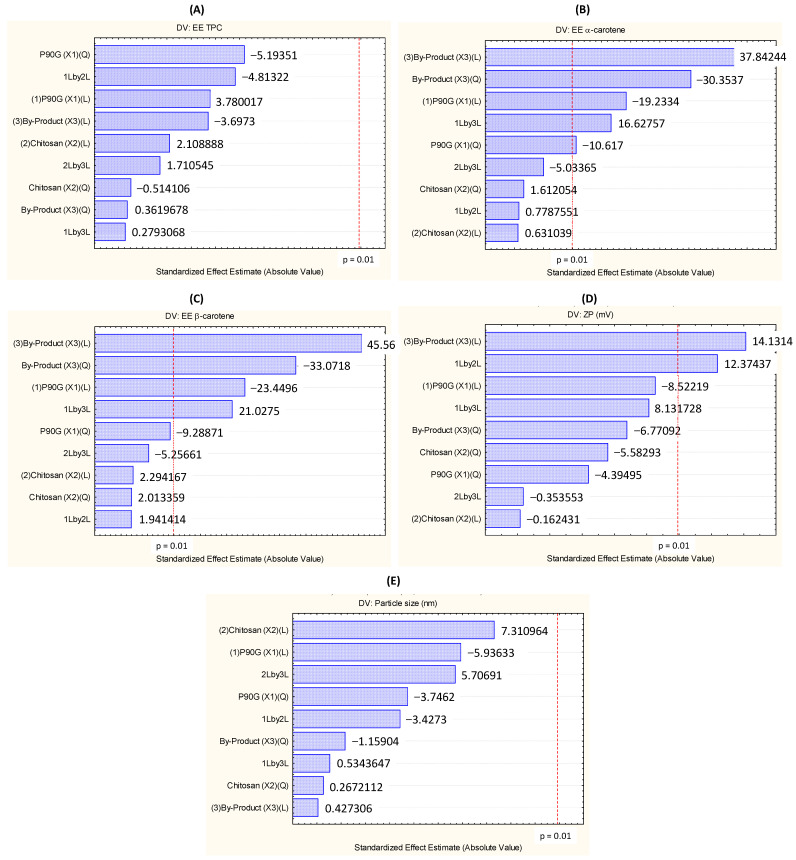
Pareto charts for the dependent variables (DV): (**A**) EE of total phenolic compounds (TPC), (**B**) EE of α-carotene, and (**C**) EE of β-carotene, (**D**) zeta potential and (**E**) particle size of TPP-chitosomes containing encapsulated phenolics and carotenoids.

Table 3 shows the results for the encapsulation efficiency (EE) of phenolics obtained according to the CCD 2^3^ design and Figure 2 shows the Pareto diagrams of the responses.

Pareto diagrams show how the independent variables (phospholipid concentration, x_1_; chitosan concentration, x_2_; and carrot pomace concentration, x_3_) affect the responses. In these diagrams, the standardized effects provide the heights of the bars, which, in turn, are arranged in descending order. The effect of each independent variable is significant the further to the right of the red line it is at the 99% confidence level of significance (*p* = 0.01) [46]. Figure 2A shows that the linear, quadratic, and interaction effects of the studied factors did not significantly influence (*p* > 0.01) the EE of phenolics. In this work, the EE of phenolics ranged from 80 to 100%, so the system efficiently encapsulated these compounds.

The ANOVA statistical method was used to evaluate the significance of the regression coefficients (linear, quadratic, and interaction) of the studied factors (x_1_, x_2_, and x_3_) and the significance of the regression mathematic model and the lack of fit of the model at a significance level of 1%. The results are presented in Table 4. As observed, the linear, quadratic, and interaction coefficients were not significant for the EE of phenolics. Thus, we were not able to derive a mathematical model for the EE of phenolics in the levels of studied factors based on the carrot pomace, chitosan, and phospholipid concentrations within the studied range at 99% confidence intervals. Consequently, we did not obtain any response surfaces and could not optimize encapsulation for this response. Notwithstanding, we achieved a considerable EE of phenolics—over 80%, which highlighted the relevance of the encapsulation of phenolics. Gibis, Ruedt, and Weiss [47] obtained similar values when they encapsulated grape seed phenolics in chitosomes (EE = 99.5%), while Esposto et al. [33] obtained an EE of phenolics of 86% when they encapsulated phenolics of carrot by-product in TPP-chitosomes.

### 3.3. Encapsulation Efficiency of α- and β-Carotene

Although the studied factors did not influence the encapsulation of phenolics, a different behavior for β- and α-carotene (Figure 2B,C) was observed: the linear and quadratic effects of the carrot pomace concentration (x_3_) significantly affected the EE of α- and β-carotene, whereas the linear and quadratic effects of the P90G concentration (x_1_) significantly impacted the EE of α-carotene, while only the linear effect of the P90G concentration significantly influenced the EE of β-carotene. The interaction between the P90G (x_1_) and carrot pomace (x_3_) concentrations affected the EE of β- and α-carotene significantly. The chitosan concentration (x_2_) did not influence the EE of the bioactive compounds. This is important because using a low chitosan concentration is more viable to decrease the cost of producing particles containing chitosan, an expensive additive in Brazil (R$ 625.00 for 50 g).

We carried out ANOVA for α- and β-carotene, to find that the chitosan concentration did not affect the EE of α- and β-carotene. The independent variables of the phospholipid concentration (x_1_) and carrot pomace concentration (x_3_), their quadratic terms, and the interaction between them (*β*_13_) significantly (*p* < 0.01) affected the EE of α- and β-carotene. We adjusted the values to the quadratic polynomial model according to the CCD 2^3^ design. The tests were significant (F_calculated_ > F_tabulated1_) and predictive (F_lack of fit_ < F_tabulated2_) for the EE of α- and β-carotene. The mathematical model considered only the significant parameters as shown in Equations (3) and (4):

EE of α-carotene = 99.68 − 2.08 × x_1_ − 1.32 × x_1_^2^ + 4.09 × x_3_ − 3.67 × x_3_^2^ + 2.35 × x_1_ × x_3_ (3)

EE of β-carotene = 99.00 − 1.88 × x_1_ + 3.65 × x_3_ − 2.78 × x_3_^2^ + 2.20 × x_1_ × x_3_ (4)

where x_1_ is the coded variable for the phospholipid concentration, and x_3_ is the coded variable for the carrot pomace concentration.

**Figure 3:**
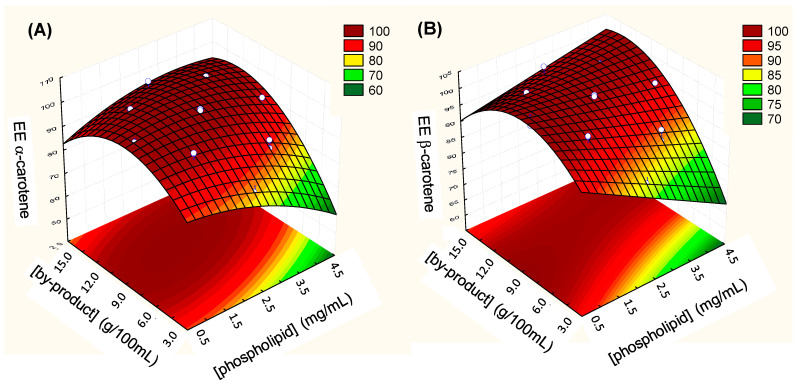
Fitted surface for the EE of (**A**) α-carotene and (**B**) β-carotene.

Based on these models, we obtained fitted surface graphs for the EE of α- and β-carotene (Figure 3). The highest EE of α- and β-carotene can be seen in the red regions, while the green regions represent a decrease in the EE. Analysis of the response surface reinforced that the intermediate carrot pomace concentrations (9 g/100 mL of ethanol) provided a higher EE than the lowest (4 g/100 mL of ethanol) and highest (14 g/100 mL of ethanol) carrot pomace concentrations. These results also indicated that the higher phospholipid concentrations negatively influenced the EE of α- and β-carotene, as experimentally observed in Run 10 (Table 3), which gave a lower EE than the other samples with the same chitosan and carrot pomace concentrations but lower phospholipid concentrations. Excess-free phospholipids in the aqueous suspension can form more uncoated liposomes, which are thermodynamically unstable [48] and may release bioactive compounds during the preparation.

### 3.4. Zeta Potential of TPP-Chitosomes Containing Encapsulated Phenolics and Carotenoids

Zeta potential indicates the particle surface charge and hence particle stability, preventing aggregates from being formed. The experimental design samples had a zeta potential varying from +17 to +37 mV (Table 3). The values were positive because protonated amino groups of chitosan predominated on the TPP-chitosome surface, confirming that liposomes were coated with the polymer. In Esposto et al. [33], the zeta potential varied between −4 mV and −2.5 mV for the liposome samples because the vesicle surface contained negatively charged groups due to phospholipids.

Our results resemble the results reported by other authors. Hassani, Laouini, Fessi, and Charcosset [49] encapsulated tacrinein TPP-chitosomes and obtained particles with a zeta potential ranging from +29.6 ± 1.2 mV to +37.0 ± 1.1 mV. Pan et al. [45] analyzed how the degree of reticulation is related to the properties of crosslinked TPP-chitosome nanoparticles and found a zeta potential of +30.5 ± 1.9 mV.

The Pareto chart demonstrated that the carrot pomace concentration (x_3_) and the interaction between the P90G and chitosan concentrations (x_1_x_2_) influenced the zeta potential of TPP-chitosomes containing encapsulated phenolics and carotenoids (Figure 2D). To evaluate how the independent variables affected the zeta potential, ANOVA with a significance level of 99% was conducted. The carrot pomace concentration affected the zeta potential positively and significantly (*p* < 0.01), indicating that higher carrot pomace concentrations should give a higher zeta potential. The chitosan concentration did not affect the zeta potential, but its interaction with the phospholipid concentration had significant and positive effects. The phospholipid concentration did not affect the zeta potential, but samples with lower phospholipid concentrations, like the sample used in Run 9 (0.8 mg/mL), had a higher zeta potential (+37 ± 3 mV) than the samples with lower phospholipid concentrations combined with lower chitosan and carrot pomace concentrations, which was the case of the sample used in Run 2 (3.5 mg/mL phospholipid, 4.0 mg/mL chitosan, and 6.0 g of carrot pomace/100 mL of ethanol), which had a zeta potential of +17 ± 4 mV.

These values were adjusted to the quadratic polynomial model according to the CCD 2^3^ design. The F test was significant (F_calculated_ > F_tabulated1_) and predictive (F_lack of fit_ < F_tabulated2_) and allowed us to obtain a mathematical model for this variable. Considering only the independent variables and interactions that were significant, the model can be seen in Equation (5).

Ζeta potential = 30.12 + 3.83 × x_3_ + 4.38 × x_1_ × x_2_ (5)

where x_3_ is the coded value for the carrot pomace concentration, and x_1_x_2_ is the coded value for the interaction between the phospholipid and chitosan concentrations.

### 3.5. Particle Morphology and Size Distribution

**Figure 4:**
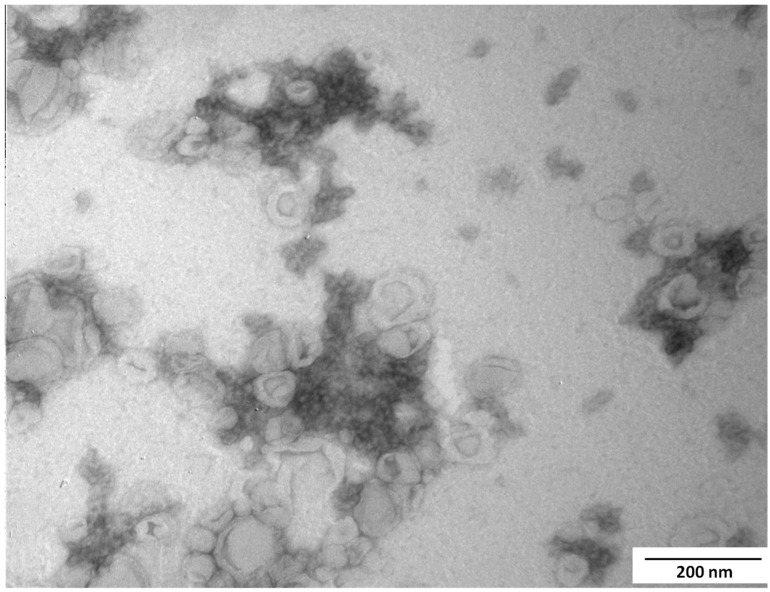
TEM micrograph of TPP-chitosomes containing encapsulated phenolics and carotenoids obtained by using the central point condition (2.5 mg/mL phospholipid, 6.0 mg/mL chitosan, and 9.0 g of carrot pomace/100 mL of ethanol). Scale bar = 200 nm.

Figure 4 presents the TEM image recorded for the central point sample. The particle structure aligned with previously reported structures [33]. The image revealed the presence of free liposomes and TPP-chitosomes containing encapsulated phenolics and carotenoids, formed by liposomes in a crosslinked chitosan network, distinguishable as dark structures. Chitosan reticulation during the preparation involved a few spherically shaped liposomes.

TPP-chitosomes containing encapsulated phenolics and carotenoids showed polymodal distribution with different average particle sizes. Table 3 presents the average diameters obtained and the relative area of the peak (%) for each population. Two main peaks were observed, at D1 and D2, which varied from 49 to 385 nm and from 306 to 748 nm, respectively, as a function of the levels of x_1_, x_2_, and x_3_ used during encapsulation. Most TPP-chitosomes containing encapsulated phenolics and carotenoids presented three populations of particles with different diameters (D1, D2, and D3), except for the samples used in Runs 1, 3, 9, and 15, which had only two populations. In general, D1 represented small diameters (54–385 nm), corresponded to uncoated liposomes (Figure 4), and accounted for a small percentage of the curve’s graphical area (~30%). This is consistent with our previous study [33], where liposomes had diameters of 60–70 nm before 11 days of storage. D2 was the diameter of the second population (522 ± 143 nm, corresponding to TPP-chitosomes containing encapsulated phenolics and carotenoids (Figure 4), and accounted for a higher percentage of the area (~67%). D3, in the micrometric scale, represented the diameter of agglomerates.

Analysis of the Pareto chart for the particle size (D1 or D2, based on the peak’s largest percentage area for each sample) showed that the independent variables and their interactions did not affect this response (Figure 2E). The model for the particle size was neither significant nor predictive (Table 4). Therefore, the results did not allow us to obtain a mathematical model for the particle size as a function of the carrot pomace, chitosan, and phospholipid concentrations within the studied range at a 99% confidence interval. Therefore, we could not obtain response surfaces or optimize encapsulation for this response.

### 3.6. Optimization of the Encapsulation Efficiency of α- and β-Carotene and Zeta Potential of TPP-Chitosomes Containing Encapsulated Phenolics and Carotenoids

**Figure 5:**
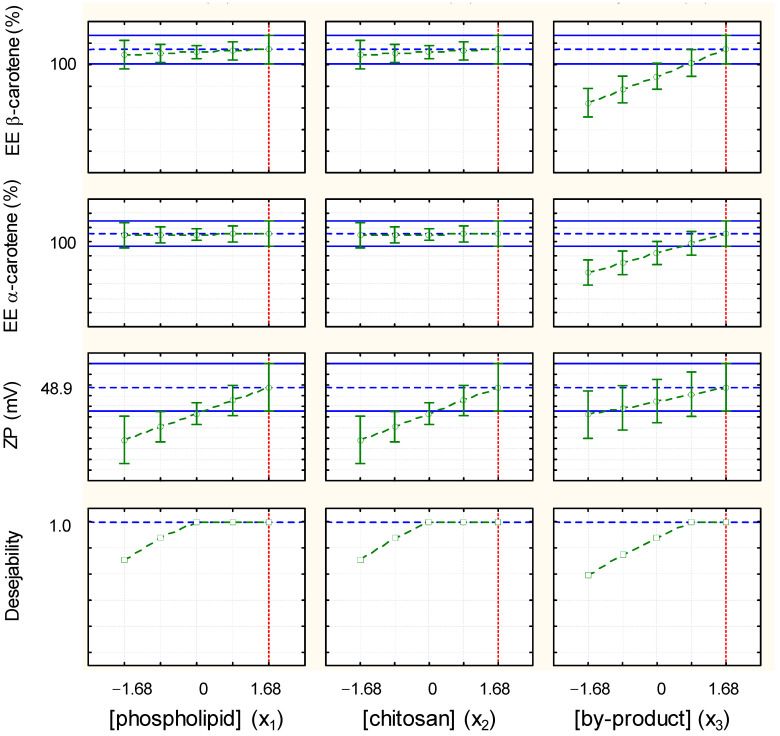
Profiles for predicted values and desirability. Blue line (horizontal) means the desirability limits for dependent predicted variables and the optimal coded values are highlighted with a dashed red line (vertical).

Figure 5 shows the graphs obtained from the desirability functions, where the optimal coded values are highlighted with a dashed red line. The optimal condition to obtain TPP-chitosomes with high contents of α- and β-carotene and a high zeta potential was a phospholipid concentration of 4.2 mg/mL, chitosan concentration of 9.4 mg/mL, and carrot pomace concentration of 14 g/100 mL of ethanol (x_1_ = 1.68, x_2_ = 1.68, and x_3_ = 1.68). However, chitosan is an expensive reagent, so future scale-up of the encapsulation process demands that less chitosan be used. The same is true for the phospholipid, which, albeit more financially viable, can still make encapsulation expensive. In addition, above 12 g of carrot pomace/100 mL of ethanol, encapsulation of α- and β-carotene reaches a limit due to solvent saturation. For this reason, we chose the coded values x_1_ = 0 for the phospholipid concentration (2.5 mg/mL), x_2_ = 0 for the chitosan concentration (6.0 mg/mL), and x_3_ = 1 for the carrot pomace concentration (12.0 g/100 mL of ethanol), which also reached the desirable limit (Figure 5) as a good condition to work with. We used the right contents of α- and β-carotene to validate the model and calculated the relative deviation (RD). We also quantified phenolics although the model obtained for this variable was not predictive. Table 5 compares the experimental and predicted values.

Because this was a predictive model, optimization of the encapsulation of α- and β-carotene was also adequate when we used intermediate values of the independent variables (2.5 mg/mL phospholipid, 6.0 mg/mL chitosan, and 9.0 g of carrot pomace/100 mL of ethanol). These particles also presented higher contents of α- and β-carotene in the extract (3.64 μg of α-carotene/mL and 9.34 μg of β-carotene/mL). The diameters D1 and D2 were 109 ± 23 nm (24%) and 502 ± 53 nm (74%), respectively, and the zeta potential was +34 ± 2 mV.

## 4. Conclusions

This study focused on encapsulating extracts derived from carrot pomace within systems consisting of liposomes coated with chitosan crosslinked with sodium tripolyphosphate polyanion (TPP-chitosomes). The resulting TPP-chitosomes containing encapsulated phenolics and carotenoids present variable size distribution and positive surface charge due to the presence of chitosan as a liposome coating. The experimental design, guided by scientific and statistical criteria, allowed us to identify several variables that influence these particles. Interestingly, the chitosan, phospholipid, and carrot pomace concentrations do not impact the EE of phenolics or particle size of TPP-chitosomes containing encapsulated phenolics and carotenoids. Conversely, these independent variables notably affect the EE of α- and β-carotene and zeta potential of the resulting particles, allowing mathematical models to be derived. Upon experimental validation, the predictions provided by the model closely match the obtained results; the relative deviation is 2%. The particles generated under optimized conditions hold potential applications across the food, cosmetic, and nutraceutical industries and primarily serve as natural pigments because they efficiently encapsulate carotenoids. They can also serve as promising vehicles for encapsulating other pigments, especially lipophilics, which can help address the challenges associated with their incorporation into aqueous suspensions.

The method of encapsulation used is particularly suitable and sustainable. The use of chitosan and TPP, both of which are biodegradable and derived from abundant natural resources, makes the process environmentally friendly. The optimization of the efficiency of the encapsulation process allows the reduction in waste and maximizes the use of bioactive compounds, contributing to cost-effectiveness. This is particularly beneficial in utilizing food industry by-products, like carrot pomace, which would otherwise be discarded, thus promoting sustainability and resource efficiency. Furthermore, knowing the most appropriate material concentrations to ensure the highest encapsulation efficiency of active compounds is important to obtain reproducible and efficient processes.

## Figures and Tables

**Table 1:** Central Composite Design (CCD) 2^3^ (8 experiments + 6 axial points + 3 central points) used to optimize encapsulation. Three variables were studied as follows: phospholipid (X_1_: 0.8, 1.5, 2.5, 3.5, and 4.2 mg/mL), chitosan (X_2_: 2.6, 4, 6, 8, and 9.4 mg/mL), and carrot pomace (X_3_: 4, 6, 9, 12, and 14 g/100 mL of ethanol) concentrations.

Sample	P90G, mg/mL (X_1_) *	Chitosan, mg/mL (X_2_) *	By-Product, g/100 mL of Ethanol (X_3_) *
1	1.5 (−1)	4.0 (−1)	6.0 (−1)
2	3.5 (+1)	4.0 (−1)	6.0 (−1)
3	1.5 (−1)	8.0 (+1)	6.0 (−1)
4	3.5 (+1)	8.0 (+1)	6.0 (−1)
5	1.5 (−1)	4.0 (−1)	12 (+1)
6	3.5 (+1)	4.0 (−1)	12 (+1)
7	1.5 (−1)	8.0 (+1)	12 (+1)
8	3.5 (+1)	8.0 (+1)	12 (+1)
9	0.8 (−1.68)	6.0 (0)	9.0 (0)
10	4.2 (+1.68)	6.0 (0)	9.0 (0)
11	2.5 (0)	2.6 (−1.68)	9.0 (0)
12	2.5 (0)	9.4 (+1.68)	9.0 (0)
13	2.5 (0)	6.0 (0)	4.0 (−1.68)
14	2.5 (0)	6.0 (0)	14.0 (+1.68)
15	2.5 (0)	6.0 (0)	9.0 (0)
16	2.5 (0)	6.0 (0)	9.0 (0)
17	2.5 (0)	6.0 (0)	9.0 (0)

* X_1_, X_2_, X_3_: encoded values in parentheses.

**Table 2:** Total phenolic compounds (TPC), α- and β-carotene contents of carrot by-product extracts according to the concentration of by-product (μg/g) or extract (μg/mL).

By-Product, g/100 mL of Ethanol (x_3_)	TPC μg GAE/g	TPC μg GAE/mL	β-Carotene μg/g	β-Carotene μg/mL	α-Carotene μg/g	α-Carotene μg/mL
4	168 ± 1	6.7 ± 0.1	129 ± 5	5.2 ± 0.1	51 ± 1	2.1 ± 0.1
6	200 ± 3	12.1 ± 0.2	116 ± 11	7.1 ± 0.1	47 ± 3	2.9 ± 0.1
9	164 ± 20	14.9 ± 1.8	96 ± 8	8.9 ± 0.7	38 ± 2	3.5 ± 0.1
12	175 ± 20	21.1 ± 2.3	57 ± 4	7.0 ± 0.3	23 ± 1	2.8 ± 0.1
14	143 ± 5	20.0 ± 0.1	49 ± 1	8.2 ± 1.3	22 ± 1	3.5 ± 0.4

**Table 3:** Results for the encapsulation efficiency (EE) of phenolics and of β- and α-carotene; hydrodynamic diameters of bi/trimodal distribution (D1, D2, D3, nm), the relative area of the peak (%), and zeta potential, of the TPP-chitosomes obtained by using the experimental conditions (CCD 2^3^) (Table 1).

Sample	Phenolics EE (%)	β-Carotene EE (%)	α-Carotene EE (%)	D1 (nm)	% Area—D1	D2 (nm)	% Area—D2	D3 (nm)	% Area—D3	ζ (mV)
1	88	98	97	53	13	339	87	-	-	+35 ± 4
2	97	87	85	111	41	442	58	-	-	+17 ± 4
3	100	98	97	64	14	547	86	-	-	+24 ± 1
4	85	90	89	164	53	719	31	3740	16	+24 ± 3
5	88	99	98	332	30	565	22	-	-	+36 ± 3
6	90	99	99	250	90			4940	10	+30 ± 3
7	92	100	98	156	31	748	69	5560	0,5	+25 ± 1
8	94	98	97	385	30	631	70	-	-	+36 ± 4
9	80	99	98	76	12	534	88	-	-	+37 ± 3
10	97	96	93	118	46	415	33	1064	17	+26 ± 2
11	97	100	100	54	9	292	91	-	-	+28 ± 1
12	96	100	100	176	46	723	52	-	-	+33 ± 4
13	100	83	80	105	37	580	62	5560	0,3	+22 ± 1
14	94	99	98	49	5	306	95	-	-	+37 ± 3
15	94	100	100	120	30	545	70	-	-	+33 ± 0
16	96	100	99	127	28	442	64	4205	8	+34 ± 2
17	93	99	99	79	14	520	87	-	-	+35 ± 2

**Table 4:** Regression coefficients and analysis of variance (ANOVA) for the EE of phenolics (TPC), α-carotene (α), and β-carotene (β) and particle size and zeta potential of TPP-chitosomes containing encapsulated phenolics and carotenoids.

Coefficient	TPC EE (y_1_)	α-Carotene EE (y_3_)	β-Carotene EE (y_2_)	Particle Size (y_4_)	ζ (y_5_)
*β*_0_	95.15	99.44	99.63	501.6	34.13 *
Linear					
*β*_1_	1.45	−2.08 *	−1.88 *	−86.3	−2.31
*β*_2_	0.81	0.07	0.18	106.3	−0.04
*β*_3_	−1.42	4.09 *	3.65 *	0.6	3.83 *
Quadratic					
*β*_11_	−2.20	−1.26 *	−0.82	−60.0	−1.31
*β*_22_	−0.22	0.19	0.18	−4.3	−1.67
*β*_33_	0.15	−3.61 *	−2.92 *	−18.6	−2.02
Interaction					
*β*_12_	−2.41	0.11	0.20	−65.1	4.38 *
*β*_13_	0.14	2.35 *	2.20 *	10.2	2.88
*β*_23_	0.86	−0.71	−0.55	108.4	−0.13
R^2^	0.49	0.94	0.94	0.79	0.92
F_calculated_	0.75	31.70	28.16	2.93	9.84
F_tabulated1_	6.72	5.32	5.41	6.72	6.51
F_lack of fit_	-	24.00	44.63		20.75
F_tabulated2_	-	99.39	99.40		99.42

* Indicates significance at 99% confidence intervals.

**Table 5:** Experimental and predicted values for the EE of α- and β-carotene.

Dependent Variable	EE of TPC (%)	EE of α-Carotene (%)	EE of β-Carotene (%)
Experimental value	95	98	99
Predicted value	-	96	97
RD (%)	-	+2	+2

RD (%): [(experimental value − predicted value)/experimental value] × 100.

## Data Availability

The original contributions presented in the study are included in the article. Further inquiries can be directed to the corresponding author.
